# Clinical efficacy of hepatic arterial infusion chemotherapy combined with transhepatic arterial embolization plus lenvatinib and tislelizumab or transarterial chemoembolization combined with lenvatinib plus tislelizumab in the treatment of advanced hepatocellular carcinoma

**DOI:** 10.3389/fonc.2025.1665348

**Published:** 2025-12-02

**Authors:** Zha Peng, Yaqiong Wang, Boyu Chen, Zhuangrong Zhu, Chengyi He, Yang Wei, Hai Huang

**Affiliations:** 1 Department of Surgery, Dongguan Hospital of Guangzhou University of Traditional Chinese Medicine, Dongguan, China; 2 Department of Hepatobiliary Surgery, Wuming Hospital of Guangxi Medical University, Nanning, China; 3 Department of General Surgery, The Second Affiliated Hospital of Guangxi Medical University, Nanning, China

**Keywords:** hepatocellular carcinoma, transhepatic arterial embolization, hepatic arterial infusion chemotherapy, transarterial chemoembolization, lenvatinib, tislelizumab

## Abstract

**Purpose:**

Advanced hepatocellular carcinoma (HCC) remains a challenge for clinical treatment. The purpose of this study is to compare the efficacy of a quadruple combination of hepatic arterial infusion chemotherapy (HAIC) with transhepatic arterial embolization (TAE), lenvatinib, and tislelizumab (THLP group) with that of a triple combination of transarterial chemoembolization (TACE) with lenvatinib and tislelizumab (TLP group) in advanced HCC.

**Methods:**

In this retrospective study, we analyzed clinical information on patients diagnosed with advanced HCC with different treatments between October 2020 and December 2022 at Wuming Hospital. Primary outcomes included overall survival (OS), and progression-free survival (PFS), objective response rate (ORR), disease control rate (DCR).

**Results:**

The study included 116 patients, 69 in the TLP group and 47 in the THLP group. Median OS and PFS for the entire cohort were 13 (95% CI, 12.0-14.0) and 9.0 (95% CI, 7.7-10.2) months. OS (median, 17 vs. 12 months, *P* < 0.001) and PFS (median, 11 vs. 7 months, *P* < 0.001) were significantly prolonged in the THLP group compared with the TLP group. After correcting for confounders using 1:1 PSM, the results remained robust. The ORR and DCR were significantly higher in the THLP group than in the TLP group according to mRECIST (ORR: 74.5 vs. 52.2%, *P* = 0.015; DCR: 87.3% vs. 71.0%, *P* = 0.040). There were no grade 4 or higher adverse events.

**Conclusion:**

The quadruple combination of HAIC, TAE, lenvatinib, and tislelizumab in the treatment of advanced HCC has a better prognosis than the triple combination of TACE in combination with lenvatinib and tislelizumab, with tolerant safety.

## 1 Introduction

The incidence of hepatocellular carcinoma (HCC) is rising worldwide (1). Patients with HCC are frequently diagnosed at an advanced stage of disease beyond potentially curative treatments, such as surgical resection, transplantation, or ablation (2). Although various treatment methods such as targeted therapy, immunotherapy, hepatic arterial infusion chemotherapy (HAIC), and transcatheter arterial chemoembolization (TACE) can improve the prognosis of advanced HCC, a single treatment modality for advanced HCC brings unsatisfactory results, and local treatment combined with systemic treatment modality is the new trend for the treatment of advanced HCC in the future (3).

The value of HAIC in the management of HCC, particularly in advanced cases and those with portal vein tumor thrombosis (PVTT), is well-documented. Its recommendation as a treatment option by both Japanese and Chinese guidelines underscores its clinical importance (4, 5). Recently, a promising treatment approach combining HAIC with lenvatinib and immune checkpoint inhibitors (ICIs) has demonstrated improved outcomes (6–9), but over 40% of patients exhibited non-responsiveness to this therapy (10). TACE is the standard of care for patients with intermediate stage HCC and has been used worldwide (11). The LAUNCH trial demonstrated that the combination of TACE and lenvatinib as first-line therapy for advanced HCC was significantly more effective than lenvatinib alone. A recent meta-analysis further revealed that the approach combining TACE, lenvatinib, and PD-1 inhibitors could significantly improve overall survival (OS), progression-free survival (PFS), objective response rate (ORR), and disease control rate (DCR) in patients with advanced HCC, without markedly increasing the risk of treatment-related adverse events of any grade (12). Transhepatic arterial embolization (TAE), via ischemic damage induced by blood inflow cessation, can facilitate tumor regression in up to 50% of patients (13). A multicenter retrospective study compared the efficacy of HAIC combined with lenvatinib and tislelizumab plus TAE versus non-combination TAE therapy in patients with unresectable HCC accompanied by PVTT. The results demonstrated that the combination group showed significantly prolonged OS (median OS: 14.1 vs. 11.3 months, P = 0.041) and PFS (median PFS: 5.6 vs. 4.4 months, P = 0.037) compared to the non-combination group. Additionally, the combination therapy achieved a higher ORR (72% vs. 52%, P = 0.039), while the incidence of adverse events was comparable between the two groups (14).

A study demonstrated that in patients with unresectable HCC, HAIC combined with chemoembolization is more effective than chemoembolization alone (15). Additionally, a retrospective study illustrated that combining HAIC with TAE enhanced PFS in patients with unresectable HCC compared to TACE alone, particularly for those at BCLC stage C (16). However, it remains unclear whether the combination of HAIC, TAE, lenvatinib, and ICIs demonstrates superior efficacy compared to TACE combined with lenvatinib and ICIs in advanced HCC. This retrospective study aims to evaluates the therapeutic efficacy and safety profile of HAIC-TAE-lenvatinib-tislelizumab combination therapy versus TACE-lenvatinib-tislelizumab regimen in advanced hepatocellular HCC, with the ultimate goal of optimizing clinical decision-making.

## 2 Materials and methods

### 2.1 Study design and patients

This retrospective cohort study included patients diagnosed with initially unresectable advanced HCC at the Department of Hepatobiliary Surgery, Wuming Hospital of Guangxi Medical University. These patients received either quadruple therapy (called the THLP group, including HAIC, TAE, lenvatinib and tislelizumab) or triple therapy (called the TLP group, including TACE, lenvatinib and tislelizumab) between October 2020 and December 2022.

Inclusion criteria were as follows: 1) age between 18–75 years; 2) radiological or pathological diagnosis of HCC according to the American Association for the Study of Liver Diseases (AASLD) Practice Guidelines; 3) concomitant macrovascular invasion and/or extrahepatic metastases (BCLC Stage C or CNLC Stage IIIa/IIIb); 4) assessment of the HCC as initially unresectable by a multidisciplinary team (MDT); 5) lack of prior therapy for HCC; 6) Child-Pugh score ≤ 7 and Eastern Cooperative Oncology Group Physical Status Score (ECOG PS)≤1; 7) received at least two cycles of interventional therapy; 9) complete medical and follow-up data available, including laboratory tests and imaging evaluations within one week prior to the initial treatment, including enhanced computed tomography (CT) and magnetic resonance imaging (MRI).

Exclusion criteria: 1) central nervous system metastases; 2) history of organ transplantation; 3) previous TACE, HAIC, radiotherapy, or systemic therapy; 4) malignancy besides HCC; or 5) severe medical comorbidities including severe cardiac, pulmonary, renal, or coagulation dysfunction.

Main portal vein invasion was defined as tumor thrombus involvement of the main portal trunk and/or its first-order branches. ALBI score = (log_12_ [bilirubin] × 0.66) + ([albumin] × -0.085), where bilirubin is in μmol/L and albumin is in g/L (17).

The study was approved by the Ethics Committee of Wuming Hospital of Guangxi Medical University and was conducted in strict accordance with the principles of the Declaration of Helsinki (NO. WM-2025(02)). Due to the retrospective nature of the study, the committee waived the requirement of informed consent. All patient-related data used in this study were de-identified and anonymized to protect privacy.

### 2.2 Treatment procedures

In the THLP group, the catheter was cannulated via the femoral artery and, depending on tumor size, location and arterial supply. The head end of the catheter was super selectively inserted into the branch of the hepatic artery supplying the tumor, and TAE was administered using emulsified poppy ethyl iodine oil. The purpose of TAE was not to completely embolize the tumor but to reduce the tumor load and to prevent excessive hepatic injury prior to HAIC. The primary embolization endpoint was a subjective angiographic chemoembolization endpoints scale (SACE) level II, indicating a reduction in antegrade arterial flow and tumor blush (18). Catheters then returned to and sheaths were left in place and returned to the ward for HAIC. The HAIC dosing regimen consisted of oxaliplatin (130 mg/m^2^ over 0–2 hours on day 1), leucovorin (200 mg/m^2^ over 2–4 hours on day 1), and fluorouracil (400 mg/m^2^, fluorouracil pushed over 15 minutes, followed by sequential infusions of 2,400 mg/m^2^ over days 1 and 2). After HAIC administration, the catheter and sheath were removed. In the TLP group, the catheter was cannulated via the femoral artery, and depending on the size, location and arterial supply of the tumor, the head end of the catheter was inserted superselectively into the branch of the hepatic artery supplying the tumor, and chemoembolization was carried out by emulsified poppy ethyl iodine oil and lobaplatin with perfusion. The end point of the embolization was the stasis in the tumor-feeding arteries. In patients with giant or bilobar multifocal lesions, in order to reduce the risk of complications, the embolization endpoint was not reached at the first TACE, but at the second or third TACE. Repeated TACE or HAIC cycles were performed every 3–5 weeks (19). The final decisions regarding the need to repeat the above treatment regimen and the exact timing are guided by the judgement of the MDT.

Lenvatinib and tislelizumab dosing regimen: daily oral lenvatinib (12 mg in patients over 60 kg and 8 mg in patients less than 60 kg) therapy was given within 7 days of the first intervention. Tislelizumab (BeiGene, Shanghai, China) 200 mg was administered intravenously every 3 weeks. Interruption or discontinuation of drug administration depends on the presence and severity of toxicity.

### 2.3 Follow-up

Patients were followed up every 4–6 weeks after initial treatment. Each follow-up visit included a detailed history, physical examination, hematological and biochemical investigations, contrast-enhanced abdominal CT or MRI, chest CT and other imaging (if clinically indicated). Final follow-up ended on 30 June 2024.

Treatment-related adverse events (TRAEs) were defined by the National Cancer Institute Common Terminology Criteria for Adverse Events (NCI CTCAE) 5.0 and grade 1 or 2 did not require modification of the treatment regimen besides promptly managed. Grade 3 or higher AEs needed to adjust treatment regimens until the AEs subsided or improved. If these AEs persisted, treatment was discontinued until they subsided. Adjustments were based on clinical judgement and residual lesion assessment. Post-embolization syndrome (manifested by fever, abdominal pain, nausea, vomiting and increased white blood cell count) and transient abnormalities of liver enzymes after embolization were expected reactions and subsided within a short period of time and were therefore not recorded separately.

### 2.4 Assessment of efficacy

OS and PFS were compared between THLP and TLP groups. OS was defined as the time from the start of treatment to death from any cause. PFS was defined as the time from the start of treatment to the first disease progression or death, whichever occurred first. Contrast-enhanced CT or MRI scans were performed every four cycles. Tumor response types were classified as complete response (CR), partial response (PR), stable disease (SD) and progressive disease (PD) according to the modified response evaluation criteria in solid tumors (mRECIST) and modified response evaluation criteria in solid tumors (RECIST) 1.1 criteria (20, 21). ORR was defined as the percentage of patients in whom CR and PR occurred. DCR was defined as the percentage of patients who experienced CR, PR and SD.

### 2.5 Statistical analysis

Continuous variables were expressed as mean ± standard deviation (SD) or median (interquartile range, IQR) and compared using the Student’s t-test or the Mann–Whitney U-test based on their distribution. Categorical variables were presented as counts and percentages, and differences between the two groups were assessed using the chi-square test or Fisher’s exact test. Survival curves were analyzed using the Kaplan-Meier method and the log-rank test. Propensity score matching (PSM) analysis was used to reduce the effect of confounding factors. A 1:1 nearest neighbor matching algorithm was applied with a caliper width of 0.02. Variables with *P* < 0.10 in the univariate analysis were included in the multivariate analysis, and independent prognostic factors for OS and PFS were determined using the COX regression analyses. All statistical tests were two-tailed and *P* < 0.05 was considered statistically significant. Data processing was performed using SPSS 25.0 (SPSS, Chicago, IL, USA) and R (version 4.0.3; R Foundation Inc., Vienna, Austria).

## 3 Results

### 3.1 Comparison of baseline information

**Figure 1:**
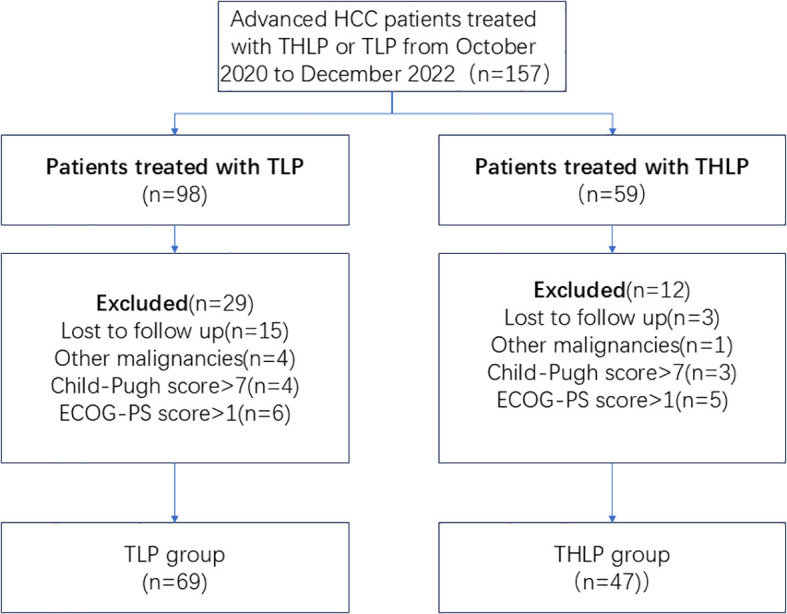
Flow diagram of patient enrollment. HCC, hepatocellular carcinoma; THLP, transhepatic arterial embolization combined with hepatic arterial infusion chemotherapy, lenvatinib plus tislelizumab; TLP, transarterial chemoembolization combined with lenvatinib plus tislelizumab; ECOG-PS, Eastern Cooperative Oncology Group Performance Status.

We assessed the eligibility of 157 patients with unresectable HCC treated with THLP or TLP. Of these, 5 had other types of malignancies, 7 patients had Child-Pugh scores greater than 7, and 11 patients had ECOG-PS scores greater than 1. 18 patients were lost to follow-up. Finally, 116 patients were included in our study, 69 in the TLP group and 47 in the THLP group (**Figure 1**). The patients in TLP group underwent a total of 300.

TACE procedures, with a median of 4 (range, 2-8). While the patients in THLP group underwent a total of 245 TAE-HAIC procedures, with a median of 4 (range,2-8). In the TLP group, the cycles of tislelizumab injection ranged from 3 to 26, with a median of 13, while in the THLP group, the cycles of tislelizumab injection ranged from 6 to 26, with a median of 17.

**Table 1** summarized the clinical characteristics of patients with advanced HCC who received THLP or TLP. The median age of the patients was 50 years (IQR: 41-58) and 86.2% of them were male. The BMI of entire cohort were 21.7 ± 3.3 Kg/m^2^. Before PSM, sex, age, BMI, prevalence of diabetes, whether they smoked or not, ECOG-PS scores, HBsAg status, ALBI grading, FIB-4, AFP, PIVKA-II, and tumor numbers, main portal vein invasion or not, hepatic vein invasion or not, extrahepatic metastasis or not, and largest tumor size were not statistically different (all *P* > 0.05), whereas significant differences in Child-Pugh classification, and whether or not they were combined with ascites were found between the two groups (*P* < 0.05). After using 1:1 PSM analysis, 33 patients were included in each groups, and there were no significant differences in clinical characteristics between the two groups (all *P* > 0.05).

**Table 1:** Baseline characteristics of the patients.

Characteristic	Before-PSM			P	After-PSM			P
	Total (n=116)	TLP (n=69)	THLP (n=47)		Total (n=66)	TLP (n=33)	THLP (n=33)	
Age, years	50(41,58)	54(39,57)	53(42,59)	0.66	49.4 ± 11.4	50.2 ± 11.0	48.8 ± 11.9	0.623
Sex, %				0.777				1
Male	100(86.2)	60(86.9)	40(85.1)		54(82)	27(82)	27(82)	
Female	16(13.8)	9(13.1)	7(14.9)		12(18)	6(18)	6(18)	
BMI, kg/m^2^	21.7 ± 3.3	21.6 ± 3.2	21.7 ± 3.2	0.964	21.80 ± 2.9	21.80 ± 2.9	21.69 ± 3.0	0.739
Diabetes, %				0.292				0.547
Yes	20(17.2)	14(20.3)	6(12.8)		14(21)	8(24)	6(18)	
No	96(82.8)	55(79.7)	41(87.2)		52(79)	25(76)	27(82)	
Smoke, %				0.995				0.438
Yes	42(36.2)	25(36.2)	17(36.2)		23(35)	10(30)	13(39)	
No	74(63.8)	44(63.8)	30(63.8)		43(65)	23(70)	20(61)	
ECOG-PS, %				0.627				0.805
0	55(47.4)	34(49.3)	21(44.7)		31(47)	16(48)	15(45)	
1	61(52.6)	35(50.7)	26(55.3)		35(53)	17(52)	18(55)	
HBsAg, %				0.833				0.786
Positive	90(77.6)	54(78.3)	36(76.6)		47(71)	23(70)	24(73)	
Negative	26(22.4)	15(21.7)	11(23.4)		19(29)	10(30)	9(27)	
ALBI, %				0.38				0.438
1	40(34.5)	26(37.7)	14(29.8)		23(35)	13(39)	10(30)	
2	76(65.5)	43(62.3)	33(70.2)		43(65)	20(61)	23(70)	
Child-Pugh, %				0.004				0.602
A	37(31.9)	15(21.7)	22(46.8)		22(33)	12(36)	10(30)	
B	79(68.1)	54(78.3)	25(53.2)		44(67)	21(64)	23(70)	
FIB-4	4.58(2.51,7.27)	4.7(2.54,7.52)	4.47(2.37,6.06)	0.527	4.3(2.3,6.7)	4.3(2.4,7.2)	4.2(2.0,6.0)	0.442
AFP, ug/L,%				0.679				0.805
<400	59(50.9)	34(49.3)	25(53.2)		35(53)	18(55)	17(52)	
≥400	57(49.1)	35(50.7)	22(46.8)		31(47)	15(45)	16(48)	
PIVKA-II, mAU/mL, %				0.631				0.319
Normal	66(56.9)	38(55.1)	28(59.6)		38(58)	17(52)	21(64)	
Up	50(43.1)	31(44.9)	19(40.4)		28(42)	16(48)	12(36)	
Ascites, %				0.016				
Yes	65(56.0)	45(65.2)	20(42.6)		37(56)	20(61)	17(52)	0.457
No	51(44.0)	24(34.8)	27(57.4)		29(44)	13(39)	16(48)	
Number of tumors, %								1
1	78(67.2)	45(65.2)	33(70.2)	0.574	46(70)	23(70)	23(70)	
≥2	38(32.8)	24(34.8)	14(29.8)		20(30)	10(30)	10(30)	
Main portal vein invasion, %				0.076				0.792
Yes	38(32.8)	27(39.1)	11(23.4)		21(32)	10(30)	11(33)	
No	78(67.2)	42(60.9)	36(76.6)		45(68)	23(70)	22(67)	
Hepatic vein invasion, %				0.842				1
Yes	53(45.7)	31(44.9)	22(46.8)		28(42)	14	14	
No	63(54.3)	38(55.1)	25(53.2)		38(58)	19	19	
Extrahepatic metastasis, %				0.317				1
Yes	94(81.0)	59(85.5)	35(74.5)		52(79)	26	26	
No	22(19.0)	10(14.5)	12(25.5)		14(21)	7	7	
Largest tumor size, cm	10.28 ± 3.33	10.2 ± 3.3	10.4 ± 3.4	0.67	10.5 ± 3.4	10.6 ± 3.4	10.4 ± 3.5	0.617

PSM, propensity score matching; THLP, transhepatic arterial embolization combined with hepatic arterial infusion chemotherapy, lenvatinib plus tislelizumab; TLP, transarterial chemoembolization combined with lenvatinib plus tislelizumab; BMI, body mass index; ECOG-PS, Eastern Cooperative Oncology Group Performance Status; HBsAg, hepatitis B surface antigen; ALBI, Albumin-bilirubin score; AFP, a-fetoprotein; PIVKA-II, protein induced by vitamin K absence or antagonist-II; IQR, interquartile range; SD, standard deviation.

### 3.2 Survival analysis

**Figure 2:**
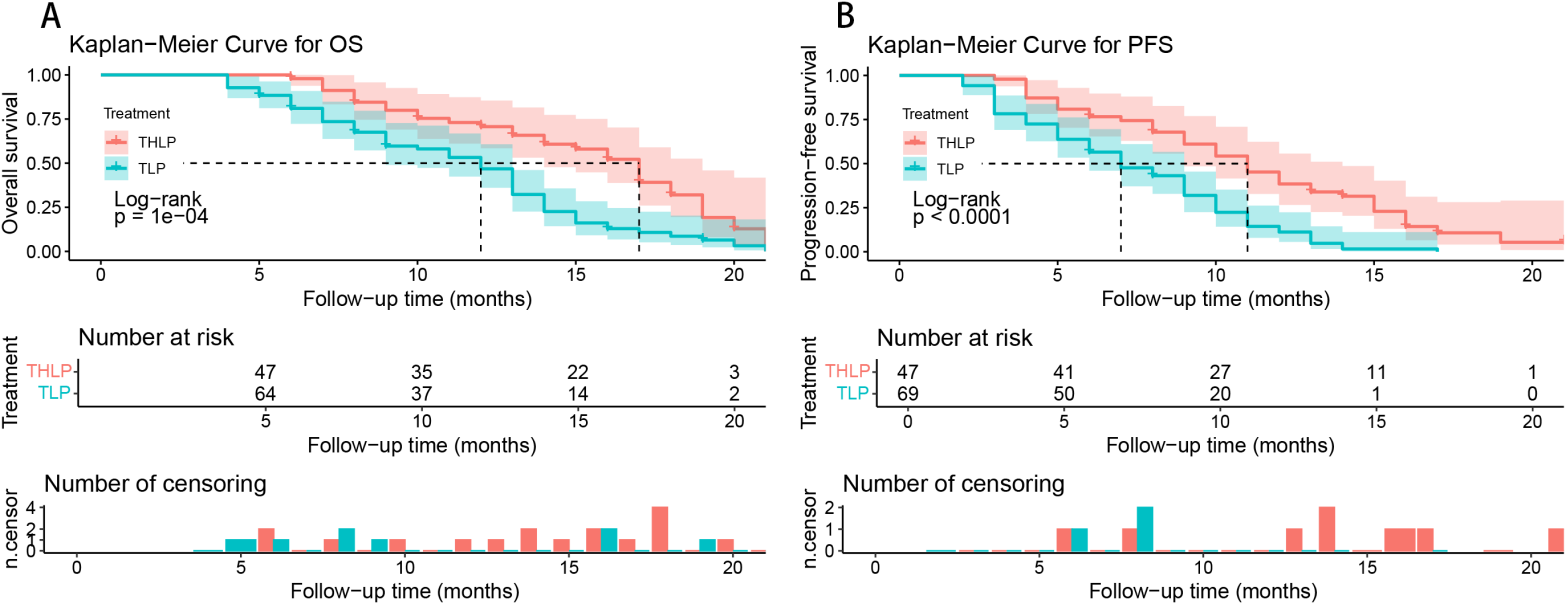
Kaplan-Meier analyses of overall survival **(A)** and progression-free survival **(B)** according to treatment groups. THLP, transhepatic arterial embolization combined with hepatic arterial infusion chemotherapy, lenvatinib plus tislelizumab; TLP, transarterial chemoembolization combined with lenvatinib plus tislelizumab.

**Figure 3:**
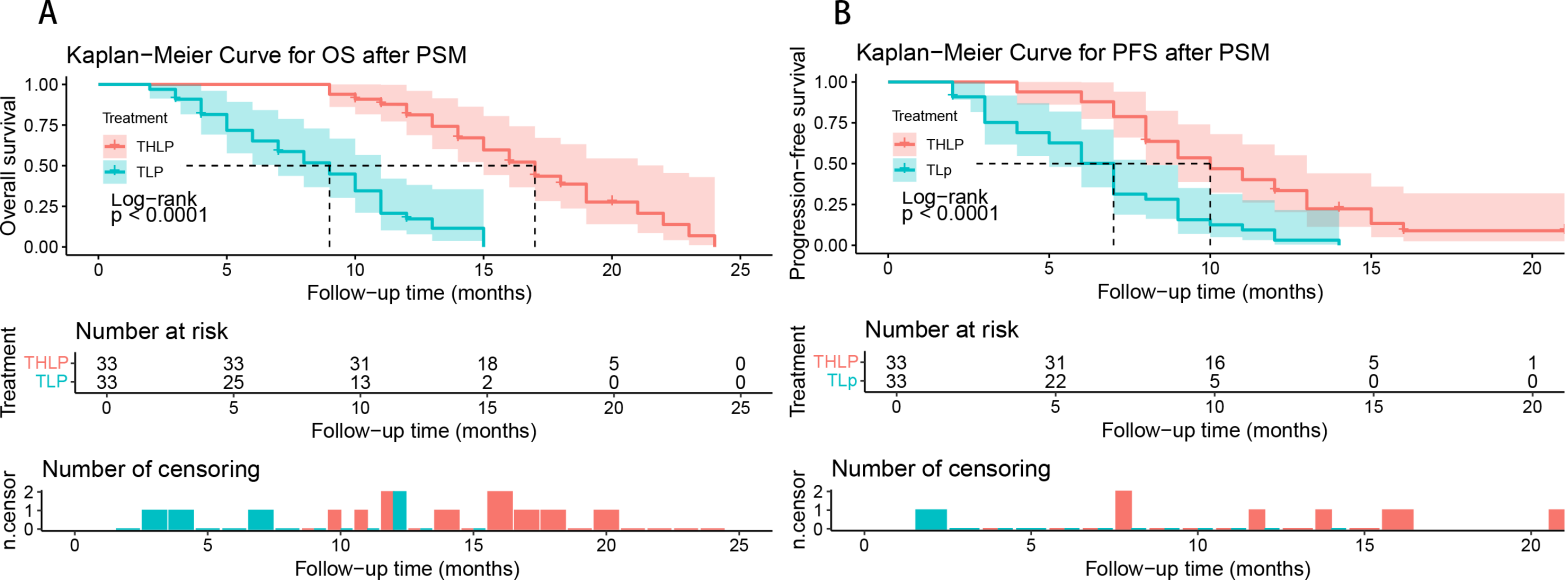
Kaplan-Meier analyses of overall survival **(A)** and progression-free survival **(B)** according to treatment groups after propensity score matching (PSM) analysis. THLP, transhepatic arterial embolization combined with hepatic arterial infusion chemotherapy, lenvatinib plus tislelizumab; TLP, transarterial chemoembolization combined with lenvatinib plus tislelizumab.

A total of 91 patients died by the follow-up cut-off date, including 30 in the THLP group and 61 in the TLP group. The median OS and PFS of all patients were 13 (95% confidence interval [CI], 12.0-14.0) months and 9.0 (95% CI, 7.7-10.2) months, respectively. The THLP group exhibited extended OS (median 17 [95%CI, 15.3-18.7] vs. 12 [95%CI, 10.6-13.4] months, hazard ratio [HR] = 2.23, 95% CI, 1.43-3.48, *P* < 0.001) and PFS (median 11 [95%CI, 9.1-12.9] vs. 7.0 [95%CI, 5.2-8.8] months, HR = 2.35, 95% CI, 1.54-3.57, *P* < 0.001) (**Figure 2**). To enhance the robustness of the results, we performed a sensitivity analysis. Since each patient completed at least two cycles of interventional therapy, with an interval of 3–5 weeks between cycles, we defined a 4-week period as the immortal time. We uniformly subtracted these 4 weeks from the starting point of the OS and PFS analyses. The results demonstrated that after adjusting for the immortal time, ​​the differences in OS and PFS between the THLP and TLP groups remained statistically significant (all *P* < 0.001) ​​(**Supplementary Figure 1**). After 1:1 PSM analysis, the THLP group still exhibited the OS (median 17 [95%CI 14.66-19.33] vs. 9 [95%CI 6.43-11.57] months, HR = 7.07, 95%CI: 3.45-14.49, *P* < 0.001) and PFS (median 10 [95%CI 7.76-12.23] vs. 7 [95%CI 5.97-8.03] months; HR = 2.88, 95%CI: 1.67-4.98, *P* < 0.001) (**Figure 3**).

### 3.3 Analysis of prognostic factors

To identify risk factors associated with OS and PFS, we performed univariate and multivariate analyses in the overall population cohort (**Table 2**). The results showed that different treatment regimens (THLP vs. TLP; HR = 1.73, 95%CI 1.06-2.83, *P* = 0.029), main portal vein invasion (present vs. absent; HR = 0.34, 95%CI 0.19-0.59, *P* < 0.001), the Child-Pugh grade (grade B vs. grade A; HR = 0.35, 95%CI 0.17-0.73, *P* = 0.005) and presence of ascites (Yes vs. No; HR = 0.38, 95%CI 0.21-0.67, *P* < 0.001) were independent prognostic factors for OS. Different treatment regimens (THLP vs. TLP; HR = 2.03, 95% CI 1.28-3.22, *P* = 0.003), main portal vein invasion (present vs. absent; HR = 0.40, 95% CI 0.24-0.65, *P* < 0.001) and presence of ascites (Yes vs. No; HR = 0.55, 95% CI 0.34-0.90, *P* = 0.017) were independent prognostic factors for PFS.

**Table 2:** Analyses of risk factors for OS and PFS of overall population.

Factor	Overall survival				Progression-free survival			
	Univariate analysis		Multivariate analysis		Univariate analysis		Multivariate analysis	
	HR (95% CI)	*P*	HR (95% CI)	*P*	HR (95% CI)	*P*	HR (95% CI)	*P*
Sex								
Male/Female	1.17(0.66,2.07)	0.595			1.10(0.64,1.90)	0.733		
Age (years)								
<60/≥60	1.26(0.72,2.21)	0.419			1.43(0.84,2.41)	0.187		
BMI								
≥18.5,≤25	Ref	Ref			Ref			
<18.5	1.35(0.78,2.33)	0.280			1.31(0.81,2.14)	0.271		
>25	1.21(0.61,2.38)	0.589			1.02(0.54,1.92)	0.960		
ECOG PS								
1/0	1.06(0.70,1.60)	0.780			1.10(0.75,1.62)	0.628		
Diabetes								
Yes/No	0.99(0.59,1.69)	0.990			1.01(0.61,1.67)	0.960		
Smoke								
Yes/No	1.03(0.67,1.59)	0.888			0.93(0.63,1.39)	0.735		
HBsAg								
Yes/No	0.99(0.61,1.05)	0.994			1.11(0.70,1.76)	0.652		
ALBI								
Grade 2/Grade 1	1.05(0.68,1.61)	0.822			1.06(0.71,1.59)	0.768		
Child-Pugh class								
B/A	0.17(0.09,0.32)	<0.001	0.35(0.17,0.73)	0.005	0.32(0.19,0.52)	<0.001	0.59(0.34,1.02)	0.058
AFP level (ug/L)								
≥400/<400	0.58(0.38,0.88)	0.01	0.72(0.46,1.13)	0.154	0.63(0.42,0.93)	0.020	0.71(0.47,1.07)	0.099
PIVKA-II (mAU/mL)								
Up/Normal	0.38(0.24,0.59)	<0.001	0.75(0.45,1.24)	0.261	0.48(0.32,0.71)	<0.001	0.75(0.49,1.16)	0.194
Ascites								
Yes/No	0.22(0.14,0.36)	<0.001	0.38(0.21,0.67)	<0.001	0.36(0.24,0.54)	<0.001	0.55(0.34,0.90)	0.017
Largest tumor size (cm)								
≥10/<10	0.82(0.53,1.25)	0.348			0.82(0.55,1.20)	0.304		
Number of tumors								
≥2/1	0.57(0.36,0.89)	0.013	0.63(0.38,1.03)	0.063	0.59(0.39,0.91)	0.016	0.68(0.43,1.08)	0.105
Main portal vein invasion								
Yes/No	0.21(0.12,0.34)	<0.001	0.34(0.19,0.59)	<0.001	0.27(0.17,0.42)	<0.001	0.40(0.24,0.65)	<0.001
Hepatic vein invasion								
Yes/No	0.64(0.42,0.99)	0.045	0.75(0.48,1.19)	0.223	0.71(0.48,1.05)	0.087	0.78(0.51,1.18)	0.241
Extrahepatic metastasis								
Yes/No	0.61(0.35,1.06)	0.081	0.89(0.47,1.71)	0.734	0.73(0.44,1.19)	0.212		
FIB-4								
>2.67/≤2.67	0.85(0.53,1.37)	0.514			1.06(0.68,1.64)	0.804		
Treatment								
THLP/TLP	2.23(1.43,3.48)	<0.001	1.73(1.06,2.83)	0.029	2.35(1.54,3.57)	<0.001	2.03(1.28,3.22)	0.003

THLP, transhepatic arterial embolization combined with hepatic arterial infusion chemotherapy, lenvatinib plus tislelizumab; TLP, transarterial chemoembolization combined with lenvatinib plus tislelizumab; BMI, body mass index; ECOG-PS, Eastern Cooperative Oncology Group Performance Status; HBsAg, hepatitis B surface antigen; ALBI, Albumin-bilirubin score; AFP, a-fetoprotein; PIVKA-II, protein induced by vitamin K absence or antagonist-II; CI: Confidence interval; HR, hazard ratio.

**Figure 4:**
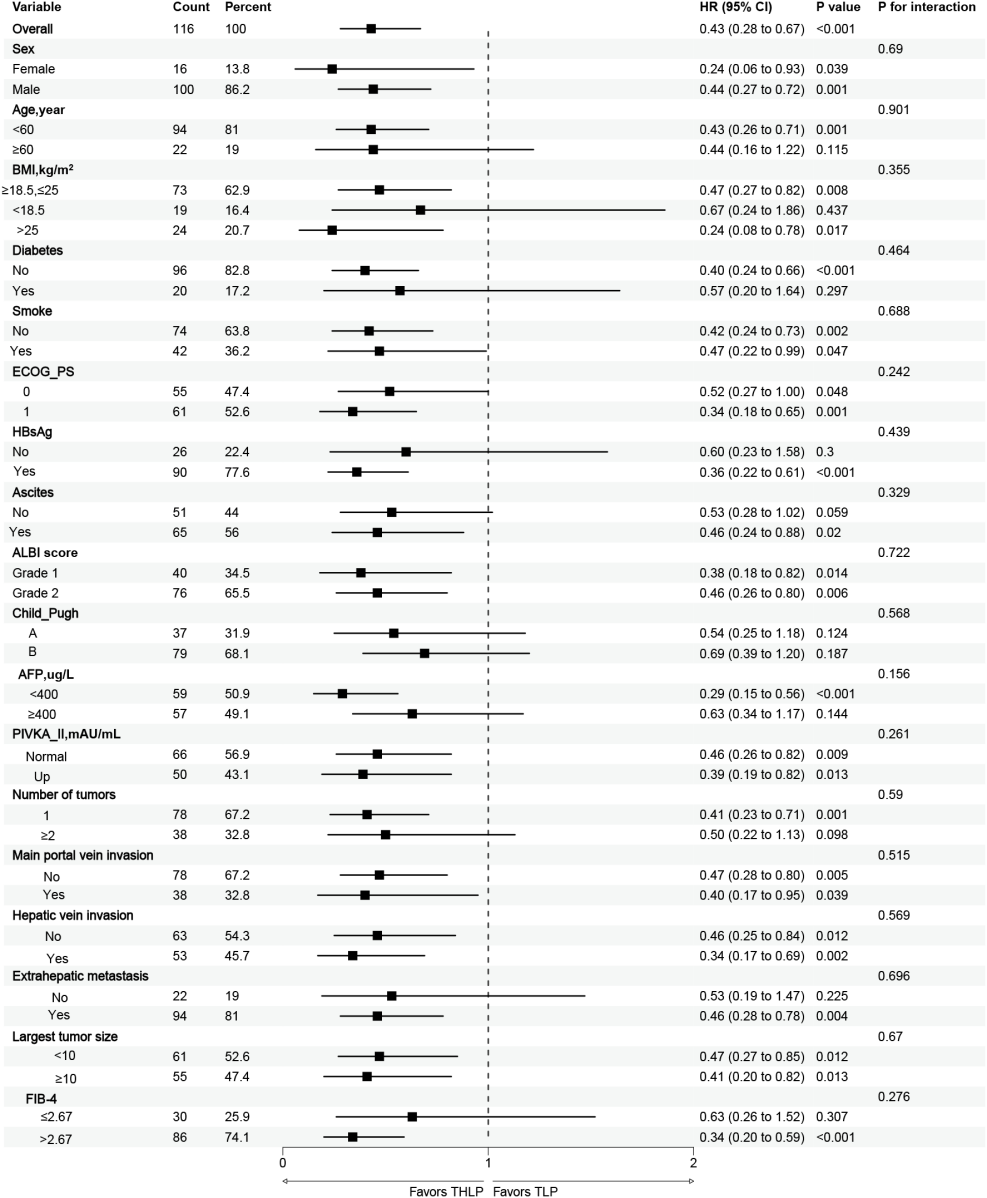
Forest plot of the subgroup analyses for overall survival. HR, hazard ratio; CI, confidence interval; THLP, transhepatic arterial embolization combined with hepatic arterial infusion chemotherapy, lenvatinib plus tislelizumab; TLP, transarterial chemoembolization combined with lenvatinib plus tislelizumab; BMI, body mass index; ECOG-PS, Eastern Cooperative Oncology Group Performance Status; HBsAg, hepatitis B surface antigen; ALBI, Albumin-bilirubin score; AFP, a-fetoprotein; PIVKA-II, protein induced by vitamin K absence or antagonist-II.

**Figure 5:**
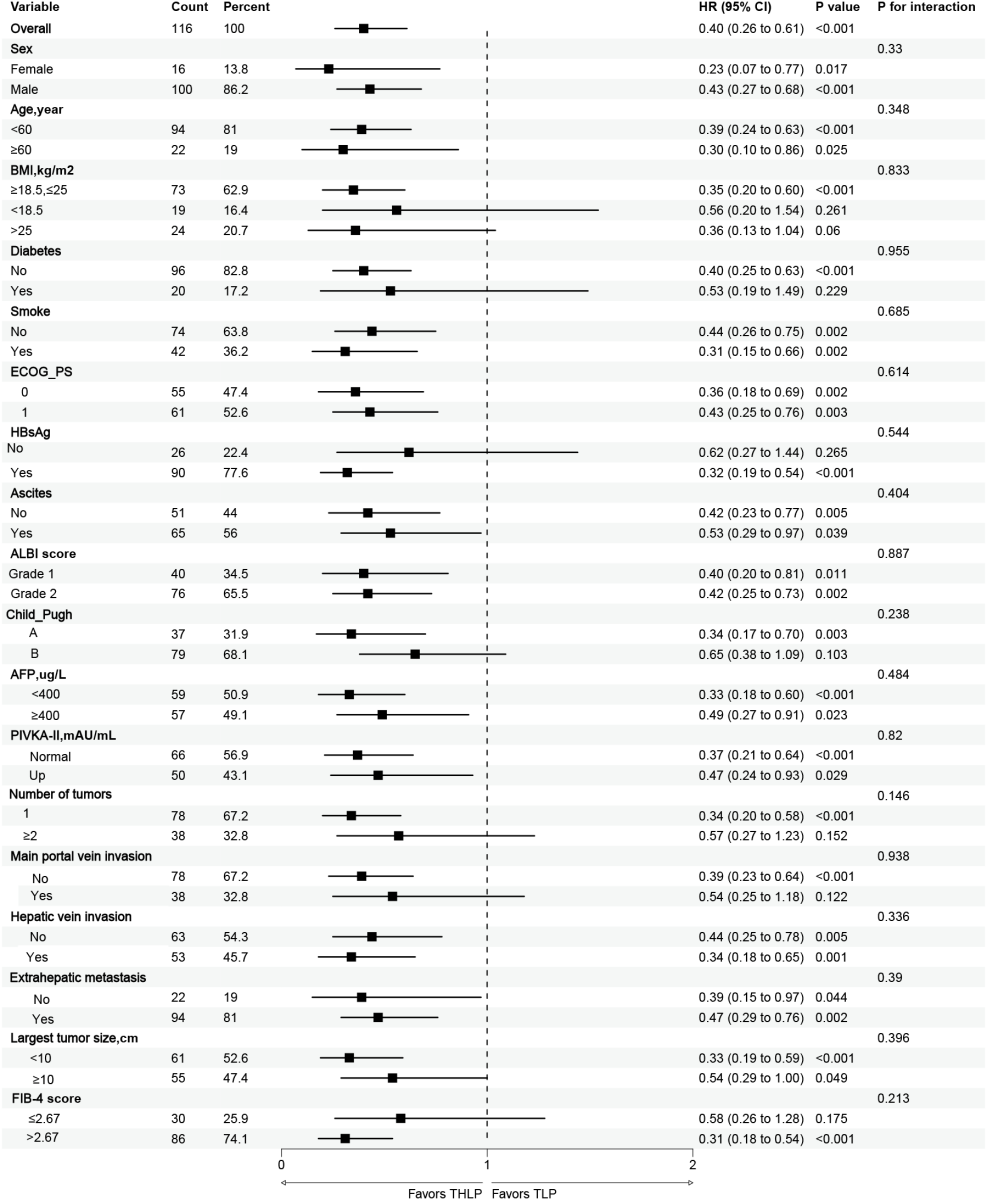
Forest plot of the subgroup analyses for progression-free survival. HR, hazard ratio; CI, confidence interval; THLP, transhepatic arterial embolization combined with hepatic arterial infusion chemotherapy, lenvatinib plus tislelizumab; TLP, transarterial chemoembolization combined with lenvatinib plus tislelizumab; BMI, body mass index; ECOG-PS, Eastern Cooperative Oncology Group Performance Status; HBsAg, hepatitis B surface antigen; ALBI, Albumin-bilirubin score; AFP, a-fetoprotein; PIVKA-II, protein induced by vitamin K absence or antagonist-II.

Subgroup analysis of OS influencing factors showed that THLP significantly prolonged OS in patients aged <60 years, with normal and BMI>25, no diabetes, ECOG-PS score of 1, HBsAg positive, with ascites, AFP ≤ 400ng/ml, single tumor, combined with extrahepatic metastases and FIB-4>2.67, but in patients with aged ≥60 years, BMI less than 18.5, combined with diabetes, ECOG-PS score of 0, HBsAg-negative, no ascites, AFP>400ng/ml, multiple tumors, and not combined extrahepatic metastases, there was no significant difference between the two therapeutic strategies in terms of change in OS (**Figure 4**). The subgroup analysis showed that THLP significantly prolonged PFS in patients with normal and BMI >25, no diabetes, HBsAg positive, Child-Pugh class A, single tumor, not combined main portal vein invasion, and largest tumor size ≤10 cm, while there was no significant difference between the two therapeutic strategies in terms of change in PFS in patients with BMI <18.5, combined with diabetes, HBsAg-negative, Child-Pugh class B, multiple tumors, with main portal vein invasion, and largest tumor size ≥10 cm (**Figure 5**).

### 3.4 Tumor responses

The tumor responses of the two groups according to the mRECIST criteria and RECIST 1.1 criteria were shown in **Table 3**. According to mRECIST criteria, PR of all patients was 61.2%, SD was 16.4% and PD was 22.4%. The ORR was 61.2% and the DCR was 77.6%. The PR was higher in the THLP group than in the TLP group (74.5% vs. 52.3%), and the SD and PD were lower than those in the TLP group (SD: 12.8% vs. 18.8%, PD: 12.8% vs. 29.0%). The ORR and DCR of THLP were higher than those of the TLP group (ORR: 74.5% vs. 52.2%, *P* = 0.015; DCR: 87.3% vs. 71.0%, *P* = 0.040).

**Table 3:** Tumor response in two groups.

Response, n (%)	mRECIST				RECIST version 1.1			
	Total (n=116)	TLP(n=69)	THLP (n=47)	*P*	Total (n=116)	TLP (n=69)	THLP (n=47)	*P*
Best overall response								
Partial response (PR)	71(61.2%)	36(52.2%)	35(74.5%)		50(43.1%)	29(42.0%)	21(44.7%)	
Stable disease (SD)	19(16.4%)	13(18.8%)	6(12.8%)		34(29.3%)	14(20.3%)	20(42.6%)	
Progressive disease (PD)	26(22.4%)	20(29.0%)	6(12.8%)		32(27.6%)	26(37.7%)	6(12.7%)	
Objective response rate (ORR)	71(61.2%)	36(52.2%)	35(74.5%)	0.015	50(43.1%)	29(42.0%)	21(44.7%)	0.777
Disease control rate (DCR)	90(77.6%)	49(71.0%)	41(87.3%)	0.040	84(81.9%)	43(78.3%)	41(87.3%)	0.003

THLP, transhepatic arterial embolization combined with hepatic arterial infusion chemotherapy, lenvatinib plus tislelizumab; TLP, transarterial chemoembolization combined with lenvatinib plus tislelizumab.

### 3.5 Safety

TRAEs were presented in **Table 4**. Although the incidence of TRAEs were high, they were manageable, with no grade 4 or higher TRAEs, and no patients experienced treatment-related deaths. Nausea, diarrhea, decreased appetite and elevated aminotransferases were the most common TRAEs. Comparison of the incidence of most adverse events between the two groups was not statistically different, only the incidence of rash and gingival hemorrhage were statistically different between THLP group and TLP group (Rash:21.3% vs. 7.2%, *P* = 0.027; Gingival hemorrhage:55.3% vs. 33.3%, *P* = 0.019); the incidence of grade 3 rash was higher in the THLP than in the TLP group (14.9% vs. 4.3%, *P* = 0.047), whereas the incidence of grade 3 gingival hemorrhage was not significantly different between the two groups (8.5% vs. 4.3%, *P* = 0.355). Although there was no significant difference in the incidence of appetite decrease, albumin and platelet decrease between the two groups, the incidence of some grade 3 TRAEs were significantly higher in the THLP group than in the TLP group (Decreased appetite:27.7% vs. 11.6%, *P* = 0.027; Albumin decrease:29.8% vs. 14.5%, *P* = 0.046; Platelet count decreased:17.0% vs. 4.3%, *P* = 0.022).

**Table 4:** Treatment-related adverse events.

Adverse events	Any grade	Grade 3/4	Any grade			Grade 3		
	Total (n=116)		TLP (n=69)	THLP (n=47)	*P*	TLP (n=69)	THLP (n=47)	*P*
Abdominal pain	31(26.7)	13(11.2)	16(23.2)	15(31.9)	0.297	5(7.2)	8(17.0)	0.101
Nausea	103(88.8)	29(25)	59(85.5)	44(93.6)	0.174	16(23.2)	13(27.7)	0.585
Diarrhea	50(43.1)	20(17.2)	29(42.0)	21(44.7)	0.777	9(13)	11(23.4)	0.147
Decreased appetite	74(63.8)	21(18.1)	41(59.4)	33(70.2)	0.235	8(11.6)	13(27.7)	0.027
Rash	15(12.9)	10(8.6)	5(7.2)	10(21.3)	0.027	3(4.3)	7(14.9)	0.047
Fatigue	12(10.3)	8(6.9)	4(5.8)	8(17)	0.051	3(4.3)	5(10.6)	0.189
Albumin decrease	33(28.4)	24(20.7)	16(23.2)	17(36.2)	0.128	10(14.5)	14(29.8)	0.046
Bilirubin increased	28(24.1)	19(16.4)	16(23.2)	12(25.5)	0.772	11(15.9)	8(17)	0.877
Aminotransferase increased	71(61.2)	34(29.3)	44(63.8)	27(57.4)	0.493	23(33.3)	11(23.4)	0.249
White blood cell decreased	75(64.7)	14(12.1)	45(65.2)	30(63.8)	0.878	7(10.1)	7(14.9)	0.441
Platelet count decreased	40(34.5)	11(9.5)	23(33.3)	17(36.2)	0.752	3(4.3)	8(17.0)	0.022
Hypertension	20(17.2)	8(6.9)	10(14.5)	10(21.3)	0.342	3(4.3)	5(10.6)	0.189
Hand-foot syndrome	19(16.4)	5(4.3)	9(13)	10(21.3)	0.239	2(2.9)	3(6.4)	0.364
Dysphonia	9(7.8)	0(0)	5(7.2)	4(8.5)	0.803	0(0)	0(0)	/
Proteinuria	7(6.0)	2(1.7)	3(4.4)	4(8.5)	0.355	0(0)	2(4.3)	0.084
Gingival hemorrhage	49(42.2)	7(6.0)	23(33.3)	26(55.3)	0.019	3(4.3)	4(8.5)	0.355
Colitis	8(6.9)	1(9.0)	5(7.2)	3(6.4)	0.857	1(1.4)	0(0)	0.407
Hypothyroidism	5(4.3)	0(0)	3(4.3)	2(4.3)	0.981	0(0)	0(0)	/

THLP, transhepatic arterial embolization combined with hepatic arterial infusion chemotherapy, lenvatinib plus tislelizumab; TLP, transarterial chemoembolization combined with lenvatinib plus tislelizumab.

## 4 Discussion

Our study demonstrated a significant survival benefit of THLP in patients with advanced HCC compared to the TLP group. The median OS was prolonged from 12 to 17 months and the median PFS was prolonged from 7 to 12 months in patients treated with THLP compared with the TLP group. There were still prolonged OS and PFS in the THLP groups even after adjustment for confounders by a 1:1 PSM. Multivariate analysis showed that the THLP regimen was an independent prognostic factor for OS and PFS in advanced HCC patients. THLP had a higher ORR and DCR compared to TLP. These results suggested that THLP may be superior to TLP for the treatment of advanced HCC.

Patients with advanced HCC usually have a poor prognosis with an expected median survival of 6–8 months due to excessive tumor burden, invasion of vital vessels or severe liver impairment (22–24). Systemic therapies, including molecular targeted therapies and immunotherapies, are the standard treatment for advanced HCC in first-line setting (25). IMBrave150 (A Study of Atezolizumab in Combination With Bevacizumab Compared With Sorafenib in Patients with Untreated Locally Advanced or Metastatic HCC) was the first trial to demonstrate substantial improvement in OS associated with the combination of atezolizumab, the programmed cell death 1 ligand 1 (PD-L1) blocking antibody, and bevacizumab, the anti-vascular endothelial growth factor A antibody, compared with sorafenib. The reported median (IQR) OS was 19.2 (17.0-23.7) months with the atezolizumab-bevacizumab combination vs 13.4 (11.4-16.9) months with sorafenib, but the ORR (measured with RECIST, version 1.1) was only 30% for the atezolizumab-bevacizumab combination compared with 5% for sorafenib (26). The RATIONALE-301 (A Randomized, Open-label, Multicenter Phase 3 Study to Compare the Efficacy and Safety of tislelizumab Versus Sorafenib as First-Line Treatment in Patients With Unresectable HCC) trial found tislelizumab, a programmed cell death 1 (PD-1) antibody, to be noninferior to sorafenib (27). Although lenvatinib did not demonstrate statistically significant superiority over sorafenib in the primary endpoint of OS, its marked advantages in the PFS and ORR have established it as a first-line treatment option for advanced HCC (28). Numerous findings suggested that lenvatinib plus a PD-1 inhibitor is a promising treatment strategy for unresectable HCC (uHCC) patients in China (29, 30). A prospective, multicenter, open-label, single arm, phase 2 study evaluating tislelizumab plus lenvatinib as first-line treatment in patients with uHCC found that confirmed ORR and DCR were 38.7% (24/62, 95% CI, 26.6–51.9) and 90.3% (56/62, 95%CI, 80.1-96.4) respectively, after a median follow-up of 15.7 months (31). Median PFS was 8.2 months (95%CI, 6.8-not evaluable) and 1-year OS was 88.6% (95% CI, 77.7-94.4) (31). The above findings suggest the necessity of exploring more effective approaches to improve the prognosis of advanced HCC. It has reported that vascular interventional therapies such as TACE and HAIC can exert synergistic anti-tumor effects with immunotherapy and targeted therapy in advanced HCC (32). In a retrospective cohort study, patients with unresectable intermediate-stage HCC beyond the up-to-11 criteria were enrolled and divided into TACE monotherapy (T), TACE combined with lenvatinib (TL), or TACE plus lenvatinib and tislelizumab (TLT) group based on the first-line treatment, respectively (33). The results showed that TLT group exhibited significantly higher ORR and DCR than the other two groups and median PFS and OS were significantly longer in the TLT group compared with the T group (PFS: 8.5 vs. 4.4 months; OS: 31.5 vs. 18.5 months; all P<0.001) and TL group (PFS: 8.5 vs. 5.5 months; OS: 31.5 vs. 20.5 months; all P<0.05) (33). Another multicenter retrospective study demonstrated that compared to lenvatinib plus tislelizumab alone, the combination of lenvatinib, tislelizumab and HAIC significantly prolonged OS and disease-free survival in patients with high tumor burden (maximum diameter of intrahepatic lesions>7 cm), with higher ORR (53.2%) and DCR (87.2%) (34). The mechanism of TACE involves embolizing tumor-feeding arteries to induce tumor necrosis, while simultaneously delivering high concentrations of chemotherapeutic agents directly to tumor cells and prolonging drug-tumor contact time (35). HAIC uses catheter technology to deliver anticancer drugs directly into the tumor via hepatic artery, which has the benefit of increasing the local concentration of anticancer drugs in the tumor and reducing systemic adverse events due to anticancer drugs (36). The principle of TAE is to reduce or terminate tumor blood flow by occluding the tumor-feeding arteries, thereby inducing tumor ischemia and ultimately leading to tumor necrosis (37). A retrospective study showed that, compared with conventional TACE, TAE + HAIC confers PFS benefit in patients with unresectable HCCs, especially those with BCLC stage C, with higher ORR (37.14%) and DCR (88.57%) (16). In our study, the combination of HAIC with TAE, lenvatinib, and tislelizumab demonstrated superior therapeutic efficacy, showing significantly greater improvements in OS and PFS compared to the TACE regimen combined with lenvatinib and tislelizumab. Furthermore, the quadruple regimen achieved an ORR of 59.6% and a DCR of 95.7%, indicating strong synergistic effects between the locoregional therapies (HAIC/TAE) and systemic agents (lenvatinib/tislelizumab).

For advanced HCC with vascular invasion or multiple intrahepatic lesions, HAIC has a clear clinical efficacy and has been recommended by the Japanese Society of Hepatology (JSH) as the first-line treatment option for HCC patients with portal vein tumor thrombus (PVTT) (4). HAIC combined with lenvatinib + PD-1 inhibitor was also superior to TACE combined with lenvatinib + PD-1 inhibitor in patients with advanced HCC combined with portal vein tumor thrombosis and portal arteriovenous fistulae (3). In this study, we found by retrospective analysis that in advanced HCC, the efficacy of the quadruple regimen of HAIC combined with TAE, lenvatinib, and tislelizumab was superior to that of the triple regimen of TACE combined with lenvatinib and tislelizumab, and that the results remained stable even after PSM analysis was used to reduce the effects of confounding variables. This may be related to the following factors: 1) Chemotherapeutic agents can not only induce apoptosis through DNA damage and cytoplasmic effects, but also induce immunogenic cell death of tumor cells. This effect can trigger an anti-tumor immune response, further promoting the efficacy of immunotherapy (38), while the induction of tumor cell death can be further enhanced by embolization of tumor feeding vessels; 2) Lenvatinib, a multikinase inhibitor with anti-proliferative and anti-angiogenic activities, can counteract hypoxia-induced angiogenesis following interventional embolization (39); and anti-PD-1 therapy can enhance the targeting of tumor cells and promotes immune attack against tumors (40); 4) TKI, when combined with anti-PD-1 therapy, produces synergistic benefits by modulating the tumor immune microenvironment and promoting tumor T-cell infiltration (41). Of note, previous studies (42, 43) evaluating the combination of TACE, lenvatinib and PD-1 inhibitors in patients with unresectable HCC reported PFS of 11.4-13.3 months and OS of 23.6-24.0 months, which appeared to be much longer than the patients treated with TLP in our study. This may be due to the fact that previous studies included a large number (25.0%-54.5%) of BCLC stage B HCC patients, who are expected to have better outcomes than the BCLC stage C patients in the present study. In addition, the larger tumor burden of the patients included in our study (tumor size of 10.28 ± 3.33 cm, with a significant proportion of patients having ≥2 intrahepatic tumors, major portal and hepatic vein invasion, or extrahepatic metastases) may contributed to the limited survival benefit of the treatment. Univariate and multivariate analyses further identified Child-Pugh class B, ascites, main portal vein invasion, and THLP as independent prognostic factors for OS. Additionally, ascites, main portal vein invasion, and THLP were confirmed as independent prognostic factors for PFS. Child-Pugh class B and the presence of ascites indicated more severe disease and poorer liver function. Portal vein thrombosis was one of the more advanced states of HCC, especially when combined with the first branch or trunk invasion of the portal vein, which called major PVTT, the prognosis was very poor (44). In our study, a subgroup analysis of main portal vein invasion found that THLP prolonged OS in patients with or without main portal vein invasion, but only prolonged PFS in patients with advanced HCC without main portal vein invasion.

In this study, both treatment regimens demonstrated favorable safety profiles. Although grade 3 AEs occurred at certain rates for each type (aminotransferase elevation: 29.3%; nausea: 25%; diarrhea: 17.2%; decreased appetite: 18.1%), no grade 4 or higher TRAEs were observed in either group. In the BGB-A317–211 study (31), lenvatinib combined with tislelizumab for uHCC showed ≥grade 3 TRAEs incidence of 28.1%. By comparison, the LEAP-002 study reported a significantly higher rate of ≥grade 3 TRAEs (63%) for pembrolizumab plus lenvatinib (29). Although the THLP group exhibited slightly higher TRAEs incidence than the TLP group in our study, most events were mild to moderate and clinically manageable. There were no grade 4 or higher TRAEs or TRAE-related deaths. We can conclude that tislelizumab plus lenvatinib combined with vascular interventional therapy maintains a favorable safety advantage. However, given the limited sample size of the current study, cautious interpretation of these results is warranted. It is worth noting that although there was no statistical difference in the comparison of the incidence of most of the TRAEs, the overall incidence of TRAEs as well as the incidence of serious TRAEs was higher in the THLP group than in the TLP group, suggesting the need for better observation and timely management in the clinic.

At our center, we consistently use poppy ethyl iodine oil as the embolic material for both TACE and TAE procedures, which is widely applied in clinical practice for arterial embolization. Ethiodized oil serves as an ideal drug carrier due to its preferential uptake by tumor tissues. Conventional TACE (cTACE) combines cytotoxic and ischemic effects by intra-arterial injection of chemotherapeutic agents concentrated in ethiodized oil. However, its long-term efficacy is limited by inconsistent drug delivery and retention. Drug-eluting bead TACE (DEB-TACE) has emerged as an alternative interventional therapy for HCC. Previous studies indicate that drug-eluting beads can prolong the release of chemotherapeutic agents and reduce their systemic concentration (45). Nonetheless, a meta-analysis involving 693 patients demonstrated that DEB-TACE did not show significant superiority over cTACE in terms of efficacy or safety (46). Moreover, patients treated with DEB-TACE experienced higher rates of liver or biliary injuries, locoregional hepatic complications, and more severe abdominal pain compared to those receiving cTACE (45). The use of the same embolic material standardizes the procedural workflow, thereby enhancing comparability between the observation and control groups.

There were several limitations to this study. First, the fact that HAIC treatment was carried out later than TACE in this unit resulted in a bipolar distribution of the included sample; second, this was a retrospective study with inherent selection bias, and PSM analysis could not completely eliminate all of these biases. Third: the total number of included cases was small, leading to a small number of cases in some subgroups in the subgroup analysis, which affected the credibility of the results; and fourth: this was a single-center study, which may have limited the generalizability of the results. However, the favorable results of HAIC in combination with TAE, lenvatinib and tirilizumab provide clues for further subsequent studies.

In conclusion, the combination of HAIC with TAE, lenvatinib and tislelizumab prolonged OS and PFS in advanced HCC patients with an acceptable rate of TRAEs compared with TACE combined with lenvatinib and tislelizumab. Multilayer combination therapy may be an ideal treatment strategy for patients with advanced HCC.

## Data Availability

The original contributions presented in the study are included in the article/**Supplementary Material**. Further inquiries can be directed to the corresponding author.
