# Supplementary figures and images for: Clinical efficacy of hepatic arterial infusion chemotherapy combined with transhepatic arterial embolization plus lenvatinib and tislelizumab or transarterial chemoembolization combined with lenvatinib plus tislelizumab in the treatment of advanced hepatocellular carcinoma
Source: Front Oncol. 2025 Dec 2;15:1665348. doi: 10.3389/fonc.2025.1665348 (PMC12706463; doi:10.3389/fonc.2025.1665348)

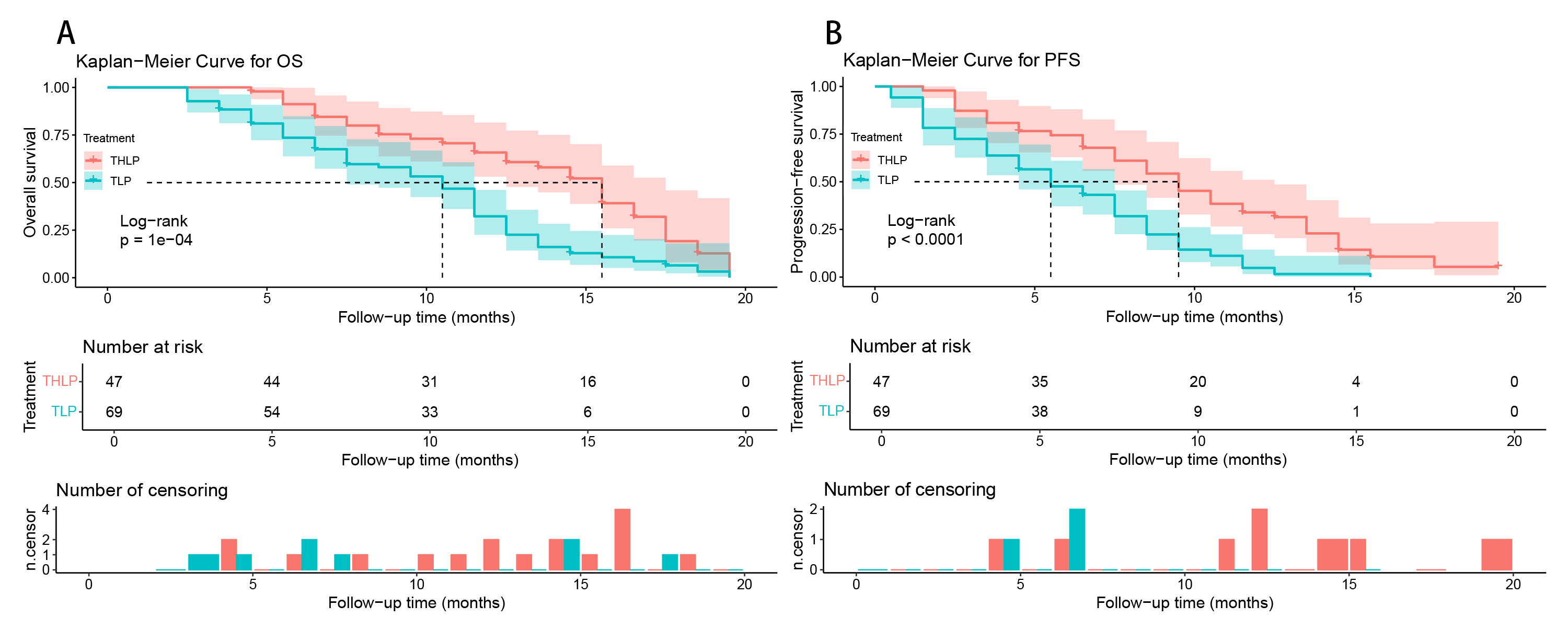

Supplement: Supplementary file 1 [file Image1.tif]
